
JOURNAL INFORMATION
==============================
NLM Title Abbreviation: Buffalo Med J Mon Rev Med Surg Sci
NLM Journal ID: 0004276

The Buffalo Medical Journal and Monthly Review of Medical and Surgical Science

Publisher: Biomedical Journal Digitization Project

ARTICLE INFORMATION
==============================
PMCID: PMC8932451
PMID: 35376948
Article version: 1
Subjects: Editorial Department

Letter detailing a case of Criminal Abortion and Infanticide

Print publication date: 1858 Dec
Volume: 14
Issue: 7
First page: 445
Last page: 447
License: This work is free of known copyright restrictions. For more information, please see PMC Back Issue Digitization.
License URL: https://creativecommons.org/publicdomain/mark/1.0/

==============================

rapid, and certain in its action than ether, and the cases of death from its administration, with proper precaution, are so rare, that we always give it the preference. We regard ether, however, as absolutely without danger. What the anæsthetic *acétone*, which is described in Brown-Séquard's Journal and which we referred to in our last number, may prove to be, we do not know; we hope that some agent may be discovered which will prove as effectual as chloroform, and as safe as ether.

The society adjourned, Nov. 4th, to meet at Mayville on the second Wednesday in May, 1859.

---

*Letter detailing a case of Criminal Abortion and Infanticide.*—We publish the following letter, which appears to have been called out by our article on *Criminal Abortions*, which details one of the most horrible instances of depravity, is rather insanity, which we have ever known. This is one of the effects of advertising abortion pills; and that such a thing should ever occur in a civilized land would be a matter of astonishment to any but the physician, who sees so much misery and crime. Cannot this matter be remedied, and will not our brother editors aid us in the effort to awaken public sentiment in this direction?

Dr. Flint—Dear Sir: I see by the two last numbers of the Buffalo Medical Journal that you have undertaken to correct and change the puhlic mind, and so do away with a great moral evil (criminal abortions). I am glad, for, one that the medical faculty are getting aroused to this great subject, and that they may show its true character and influence, and correct, if possible, the error. It is a great evil, and there must be measures and means advanced to check it, or where will it end?

I will give you the history of a case that occurred to me in my practice only a year ago.

Mrs. W., aet forty, widow woman, with three children, applied at my office for medical aid; said she had been unfortunate, and got caught in a bad scrape, and wished something to produce the desired effect. She was told that it was very dangerous to give such medicine, and that they were very uncertain in their effects, as no specific medicine was known that would produce this effect without danger. She left the office, and nothing more was heard from her for about two weeks, when I was summoned to her house as soon as I could get there. I was some twelve hours before I arrived, and when I did, I learned a tale that makes my blood almost chill when I think of it. She had procured some drug. the character of which she refused to tell; had taken it and it had the desired effect, *i. e.*, to produce abortion. She told me that she was alone with her children, who were asleep. She took the medicine, and the child was born; that she knew not what to do with it, and for fear that it would cry and awaken the other children she muffled its mouth, got off her bed, walked to the stove, in which there was a brisk fire, removed the griddle or cover, and placed the child upon the burning coals, and there it remained until it was burned to ashes. She said it writhed and tried to cry, and she had to turn away!

To what extent of crime will this debasing practice lead our mothers and our daughters if not stopped? The fœtus was a seven and a half month. The effect of the medicine which she took produced her death some hours after. This was her confession.

I could relate other instances that have occurred in my neighborhood, but they are no more than have occurred to you and to every other medical man.

I will state what I know of some of the "*Royal French Physicians*" who advertise in almost every paper that we pick up, a specific remedy for suppression of the menses, &c. You doubtless have seen an advertisement of Dr. Charles S. Gourander, French Physician, Surgeon and Accoucher, of the celebrated school of Paris, &c. Also, J. B. Bartonie, with an advertisement of like character.

These two eminent physieians are only one person assuming these two names. I am personally acquaintad with this eminent humbug and do know that he never read a medical book in his life, neves attended a medical school, never heard a medical lecture, and yct he advertised as being a graduate of the Royal College of Paris, (what students she must turn out.) I have seen this man go to the post-office and get from three to fifteen letters at a time directed to him at N. Y., and forwarded to R., and from each of these letters he received one dollar with directions to forward to such and such places his pills with full directions for use.

The most of these letters were from unmarried ladies—at least they were signed Miss So and So. These letters I have both seen and read. The pills he used were Wright's Indian Vegetable. He bought them by the gross, tore off the wrappers and put on his, and forwarded them to different parts of the United States, with directions for use. This will show the imposition practiced upon the people; but I think that his pills may be safer and less hazardous to life than the most of preparations used for this purpose, and by just such quacks as he.

Yours, &c., G. H. C.

---

*The North American Medical Reporter.* Edited by W. Elmer, M. D.

This is the title of a new journal published by W. A. Townsend & Co., New York, the publishers of Braithwaite's Retrospect. It is to be regretted that a journal which is evidently designed to be issued in a kind of connection with such a work as Braithwaite's should not take a more dignified position than the one before us. It is designed to present an index of the interesting articles which appear in the various periodicals to which it has access; so far it is good; but in addition, even the first number, which it would be policy to keep free from every thing objectionable, we regret to say, contains many things which the regular profession cannot but condemn. We should not have mentioned this publication, had it appeared under the guise of an eclectic or homeopathic journal, but the position which it aspires to assume, the circulation which it hopes to obtain among the regular profession, and especially its apparent connection with the reprint of Braithwaite's Retrospect, make it necessary for us to speak of its claims on the profession. A journal which contains a favorable notice of "The American Family Physician or Domestic Guide to Health," by John King, M. D., Professor of Obstetrics and Diseases of Women and Children in the *Eclectic* College of Medicine, Cincinnati; and the "Pronouncing Medical Lexicon," by C. H. Cleveland, M. D., Professor in the same institution, can not demand our support or sanction. We say nothing of an editorial article which reads as though it had been copied from the advertising sheet of a daily paper, announcing a new hair invigorator, the formula "to be found in a subsequent part of this number, which constitutes one of the best if not the only *rational* preparation for restoring the hair to its original or healthful condition." These are undignified and professional, and we cannot commend a publication which, like this, either panders to the interests of an eclectic, or the discoverer of a hair wash, or puts himself on a level with them, and indorses their opinions.
